# Sex Moderates Amyloid and Apolipoprotein ε4 Effects on Default Mode Network Connectivity at Rest

**DOI:** 10.3389/fneur.2019.00900

**Published:** 2019-08-20

**Authors:** Jessica Z. K. Caldwell, Xiaowei Zhuang, MacKenzie J. Leavitt, Sarah J. Banks, Jeffrey Cummings, Dietmar Cordes, The Alzheimer's Disease Neuroimaging Initiative

**Affiliations:** ^1^Cleveland Clinic Lou Ruvo Center for Brain Health, Las Vegas, NV, United States; ^2^Department of Neurosciences, University of California, San Diego, San Diego, CA, United States; ^3^UNLV School of Allied Health Sciences, Las Vegas, NV, United States

**Keywords:** aging, degenerative disease, functional MRI, memory, positron emission tomography (PET)

## Abstract

Women are more likely to have Alzheimer's disease (AD) and decline more rapidly once diagnosed despite greater verbal memory early in the disease compared to men—an advantage that has been termed “memory reserve.” Resting state functional MRI (fMRI) investigations demonstrate interactions between sex and AD risk factors in default mode network (DMN) connectivity, a network of brain regions showing progressive dysfunction in AD. Separate work suggests connectivity of left prefrontal cortex (PFC) may correlate with more general cognitive reserve in healthy aging. It is unknown whether left prefrontal functional connectivity with anterior and posterior default mode network (aDMN, pDMN) might differ by sex in AD. This study employed group independent component analysis (ICA) to analyze resting state fMRI data from 158 participants from the Alzheimer's Disease Neuroimaging Initiative (ADNI) with baseline diagnoses of normal cognition or early mild cognitive impairment (eMCI). pDMN and aDMN were defined on a subject-specific basis; prefrontal areas were selected from the Brodmann atlas (BA 6, 44, 8, and 9). Moderation regression analyses examined whether sex and amyloid PET positivity (A+/–) moderated effects of apolipoprotein ε4 (*APOE* ε*4*) on connectivity between left PFC, aDMN, and pDMN; and between aDMN and pDMN. Significant analyses were followed up with partial correlations assessing relationship of connectivity to verbal memory on the Rey Auditory Verbal Learning Test (RAVLT), and with preliminary analyses within NC and eMCI groups separately. Results showed no sex moderation of effects of A+ and *APOE* ε*4* on left prefrontal/DMN connectivity in the full sample. However, sex significantly moderated impact of A+ and *APOE* ε*4* on connectivity between aDMN and pDMN (*p* < 0.01). Women with an *APOE* allele (ε4+) and A+ showed greater aDMN/pDMN connectivity than their ε4- counterparts. No significant results were observed in men. Subgroup analyses suggested the aDMN/pDMN finding was true for those with NC, not eMCI. Partial correlations controlling for age and education showed increased aDMN/pDMN connectivity related to better verbal learning in women (*p* < 0.01) and not men (*p* = 0.18). In women at risk for AD or in early symptomatic stages who also have evidence of amyloid burden, stronger aDMN/pDMN connectivity may support verbal learning.

## Introduction

Alzheimer's disease (AD) is a progressive neurodegenerative disease that causes characteristic memory decline. Current estimates are that 5.7 million people in the US have AD, and ~2/3 of those with the disease are women (1). These statistics have led to a resurgence of research in sex-based differences in AD (2). Much of this work has delineated ways in which women show vulnerabilities and more precipitous decline due to AD (3, 4). However, a number of investigations have shown that women have some early resilience to AD-related changes, and specifically that their verbal memory remains intact despite positive amyloid positron emission tomography (PET) scan evidence of brain amyloidosis (5, 6), mild to moderate changes in hippocampal volumes (7), and reduced brain fluorodeoxyglucose (FDG) PET activity (8). This initial maintenance of memory despite pathological burden has been called memory reserve, and has been thought to reflect women's lifetime history of stronger verbal memory when compared to men (9).

Moving beyond sex-based memory reserve, a separate body of literature has delineated ways in which general cognitive reserve—often indexed by higher education levels or intelligence, and presumed to involve cognition broadly—can delay onset of AD symptoms (10). Similar to the sex-focused literature showing that women ultimately decline faster, literature on cognitive reserve in AD has shown that some individuals have a more rapid decline after an extended period of reserve-related stability (11).

The underlying neural correlates of the established concept of cognitive reserve and the newer proposal of women's memory reserve remain incompletely understood. With specific respect to women's memory reserve, work from our group suggests that compared to men, women at risk for AD may have measureable differences in volume of the hippocampus (12), a region known to be critical for both memory and AD (13). However, the hippocampus is only one potential contributor. It is possible that women's early memory resilience relates more to structural integrity of cortical networks important for memory storage and retrieval or to differences in neural function rather than structure.

In line with this, research in aging and mild cognitive impairment (MCI) suggests that a potential neural correlate of general cognitive reserve is functional resting state connectivity of the left prefrontal cortex—both globally (14, 15) and specifically with the brain's default mode network (DMN) (16). The DMN is a network of brain regions shown to be more active when people are not engaged in a particular cognitive task, i.e., at “rest” (17, 18). Brain regions connected by the DMN are among the first to be implicated in AD, and spread of AD pathology follows the DMN (19, 20). Longitudinal changes in DMN function related to AD have been delineated, such that individuals at risk or in very early stages of AD show dysfunction in posterior aspects of the DMN (pDMN) (21–25), and as AD progresses, the pattern shifts to predominant dysfunction of the anterior DMN (aDMN). This pattern has been hypothesized to reflect progressive network overload and failure (26). Interactions of sex and AD risk factors (i.e., apolipoprotein ε4 status; *APOE* ε*4*) have also been observed in the DMN, with healthy older women with an ε*4* allele showing decreased posterior DMN connectivity compared to non-carriers (27).

The left prefrontal cortex (PFC) is a large region comprised of subregions with distinct functionality and connectivity. Generally, verbal functions including verbal memory lateralize to the brain's left hemisphere in most individuals, highlighting the potential importance of this hemisphere when considering women's verbal memory reserve (28). Of particular interest to the general cognitive reserve literature are left lateral PFC (i.e., Brodmann area 6 and 44) (15, 16), and left dorsolateral prefrontal cortex (dlPFC; including BA 8 and 9). BA 6 and 44 have been shown in resting state functional connectivity studies to have anticorrelation—or negative correlation—with the DMN, with maintenance of anticorrelation marking cognitive reserve in amnestic MCI (15). The dlPFC has been implicated as a site of cognitive reserve-based structural integrity (29) and compensatory activation, such that maintained functionality on FDG PET relates to maintained cognition (30). Of note, many other brain regions have been suggested to play a role in general cognitive reserve, including right prefrontal cortex (31, 32) and inferior and middle temporal cortex (33).

No studies to date have examined whether resting state functional connectivity of left prefrontal cortex with aDMN and pDMN might serve to support women's memory reserve in AD. The present analysis sought to follow the demonstrated importance of looking at sex in AD by evaluating sex differences in the functional connectivity between left prefrontal cortex and aDMN and pDMN, and between aDMN and pDMN, in individuals with normal cognition (NC) and early mild cognitive impairment (eMCI), who were participants in a large, national study of AD (the Alzheimer's Disease Neuroimaging Initiative or ADNI) and either had or did not have known AD risk factors (i.e., *APOE* ε*4* allele) and pathological burden (i.e., positive amyloid PET). Due to reduced power, a secondary and preliminary analysis examined connectivity in the diagnostic subgroups separately, as to date findings of women's memory reserve have been at the NC stage. We specifically hypothesized that sex would moderate the effects of the presence of an *APOE* ε*4* allele and positive amyloid PET on functional connectivity within the DMN and between left prefrontal cortex and the DMN, and that this altered connectivity would relate to women's stronger preserved memory abilities.

## Materials and Methods

### Participants

Data were included from 158 participants from the ADNI (http://adni-info.org) who had available resting state functional magnetic resonance imaging (fMRI) data and were diagnosed at baseline as having normal cognition [including those with subjective memory complaints (SMC)] or eMCI. As described on the ADNI website, ADNI defined Normal cognition as no SMC, Mini Mental State Exam [MMSE; (34)] of 24–30, Clinical Dementia Rating (CDR) (35), and memory box = 0, education-adjusted raw scores on the Wechsler Memory Scale Logical Memory II test (Raw ≥ 9 for >16 years education; ≥5 for 8–15 years education; ≥3 for 0–7 years education), and no significant impairment in cognitive functions or activities of daily living. Criteria for SMC were the same as for normal cognition, but with the addition of self-reported memory problems on the Cognitive Change Index (>16). In the present analysis, individuals with SMC were combined with NC. Early MCI was defined by ADNI as SMC, MMSE of 24–30, CDR of 0.5, CDR memory box of ≥0.5, education-adjusted raw scores on the Wechsler Memory Scale Logical Memory II test (Raw = 9–11 for >16 years education; 5–9 for 8–15 years education; 3–6 for 0–7 years education), and not meeting criteria for dementia (36). Individuals with two copies of the apolipoprotein ε4 allele (*APOE* ε*4*/*4*) were excluded. Analyses were conducted in the full sample, and repeated excluding individuals with eMCI.

The study was approved by each participating ADNI site's local Institutional Review Boards, as documented on the ADNI website. All participants gave written, informed consent.

### Verbal Memory Assessment

Verbal learning and memory were assessed using the Rey Auditory Verbal Learning Test (RAVLT) (37). Specifically, we analyzed total learning and delayed free recall scores.

### Resting State fMRI Processing

For each subject, the first available resting-state fMRI scan was used for the analysis.

The first five time frames (15 s) were removed to allow the MR signal to achieve T1 equilibrium. Remaining time frames were slice-timing corrected, realigned to the mean echo-planar image using SPM12 (http://www.fil.ion.ucl.ac.uk/spm/), co-registered to the subject T1 space, normalized to the standard MNI-152 2 mm-template using Advanced Normalization Tools software (http://stnava.github.io/ANTs/) and then spatially smoothed using a 3D Gaussian filter with full-width-half-maximum (FWHM) equals 8 mm. The T1 image for each subject was segmented into gray matter, white matter and cerebrospinal fluid (CSF) to generate subject specific white matter and CSF masks. These masks were further normalized to the standard MNI-152 2 mm space. Signals from subject white matter and CSF (average time series within white matter and CSF masks), as well as six head motion parameters were regressed out from each dataset. All voxel time courses were further band pass filtered (0.008 Hz < f < 0.1 Hz) and variance normalized.

The root-mean-square (RMS) motion was computed for each subject (38). Specifically, rotational displacements were converted to translational displacements by projection to a surface of a 50 mm radius sphere and RMS head motion was then computed from both the original translational displacements and the converted rotational displacements. All subjects had less than a voxel-size RMS motion (0.30 ±0.23 mm).

**Figure 1:**
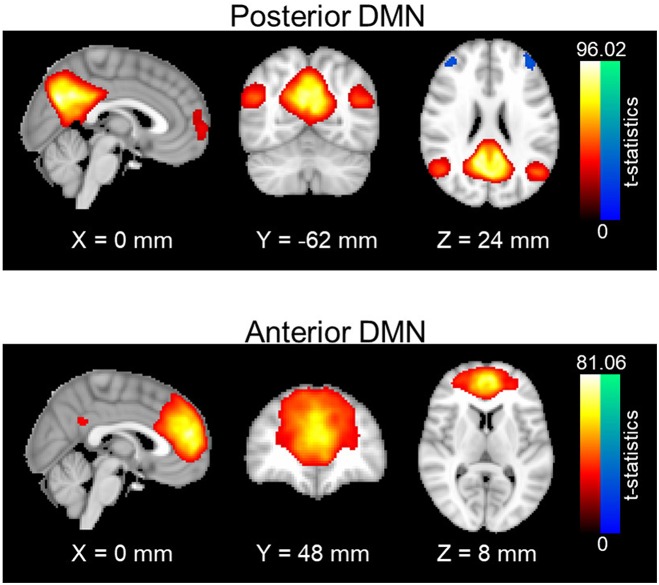
Spatial maps of anterior and posterior DMN. One-sample t-statistics have been computed across the single-subject spatial maps and thresholded at *t* = 2. DMN, Default mode network; std, standard deviation.

Resting-state networks were obtained through a spatial group independent component analysis (ICA). Principle component analysis (PCA) was first carried out for data reduction and the first 100 PCA components were retained for each subject. Data from all subjects were concatenated in time and input to the group ICA program. The ICA was carried out with in-house MATLAB scripts using the fast-ICA algorithm (39), repeated 20 times to output stable components; 30 stable ICA components were finally obtained. Out of 30 ICA components, posterior and anterior DMN were manually identified and selected (see Figure 1). Posterior DMN mainly includes posterior cingulate cortex, precuneus, bilateral angular gyrus, and bilateral middle temporal gyrus, whereas anterior DMN majorly consists of superior frontal gyrus, superior medial gyrus and anterior cingulate cortex Subject-specific spatial maps and time-courses for these two networks were reconstructed using GIG-ICA (40). Prefrontal areas were selected from the Brodmann atlas, as left Brodmann Areas (BA) 6, 44, 8, and 9. For each subject, average time courses were computed for each BA area. Functional connectivity between each prefrontal area and the DMN pair were accessed using the Pearson's correlation value between the average time courses in each BA area and the subject-specific network time series. Fisher z-transformation was then applied to the Pearson's correlation values so that computed *z*-values are normally distributed.

### Florbetapir PET Image Processing

Amyloid status (A+/–) was determined from the PET study most proximal to participant fMRI scan date. The summarized standardized uptake value ratio (SUVR) normalized to the cerebellum were obtained from the ADNI database and the amyloid status (A+/–) was further determined using a 1.11 cut-off.

### APOE Genotype

APOE genotype was downloaded from the ADNI website. After exclusion of individuals with two copies of the ε4 allele, a binary variable was created, coding individuals as either ε4+ or ε4−.

### Statistical Methods

Analyses were performed using Statistical Package for the Social Sciences (SPSS) and the Process Macro (41, 42).

*T*-tests or Mann–Whitney *U*-tests (the latter where group sizes differed) were used to explore group differences in demographic variables.

Moderation regressions examined whether sex and amyloid PET positivity (A+/–) moderated effects of a single copy of the *APOE* ε*4* allele on connectivity between left prefrontal cortex regions of interest (i.e., BA 6, 44, 8, and 9) and the posterior and anterior default mode network (pDMN; aDMN), as well as between aDMN and pDMN. For all analyses, connectivity was assessed as using the Fisher z transformed correlation values, *APOE* ε*4* carrier status was treated as an independent variable, A+/– as a moderator, and sex as a secondary moderator. Age at screening visit and education were included as covariates.

Significant moderation regression analyses were followed up with correlation analyses assessing the relationship of connectivity values with performance on the RAVLT immediate and delayed recall, partialling out effects of age and education.

Significant moderation regression analyses were also followed up with preliminary analyses in the NC and eMCI groups separately. All regressions and correlations were repeated; data are considered preliminary due to reduced power.

Significant models were corrected for multiple comparisons within each model using a Bonferroni correction based on the number of comparisons of interest in the model for the hypothesized three-way interaction. We are only interested in the following four comparisons: (1) A+ vs. A– *APOE* ε*4* carrying women; (2) A+ vs. A– *APOE* ε*4* carrying men; (3) A+/*APOE* ε*4* carrying women vs. A+/*APOE* ε*4* carrying men; and (4) A–/*APOE* ε*4* carrying women vs. A–/*APOE* ε*4* carrying men. Therefore, the corrected *p*-value threshold is *p* = 0.05/4.

## Results

### Demographics and Descriptives

Demographics and sex differences in demographic factors and memory for the full sample are summarized in Table 1. Demographics are additionally broken down by diagnosis in Tables S1, S2, and diagnosis and amyloid status in Table S3. Descriptive statistics for functional correlations (Pearson's *r* values) between left prefrontal and DMN and between aDMN and pDMN are summarized in Table S4, broken down by sex, diagnosis, amyloid status, and *APOE* ε*4* status.

**Table 1:** Participant demographics and group differences by sex (NC + MCI).

	**Male**	**Female**	**Group difference (*p*-value from *T*-test)**
Number of subjects	79	79	
Amyloid status	33 positive	38 positive	0.427
APOE4	24	26	0.734
Age	76.54 ± 6.57 Range: 65.6–95.4	74.15 ± 7.76 Range: 56.4–95.8	0.039
Handedness (right/left)	72/7	72/7	1.000
Years of education	16.70 ± 2.43	15.81 ± 2.70	0.031
RMS motion (mm)	0.31 ± 0.24	0.27 ± 0.22	0.180
RMS motion (mm)	0.31 ± 0.24	0.27 ± 0.22	0.180
RAVLT immediate	38.59 ± 11.09	44.65 ± 11.24	0.001
RAVLT delay	5.33 ± 3.97	6.75 ± 4.51	0.041

*APOE4, Apolipoprotein ε4 allele; RAVLT, Rey Auditory Verbal Learning Test; RMS, Root Mean Squared*.

*p-values reflect significance of Mann-Whitney tests*.

### Sex Does Not Moderate Left Prefrontal/DMN Connectivity Across Diagnoses

Moderation regression models for the aDMN showed only one significant result, for the overall model predicting aDMN connectivity with BA44 (*p* = 0.05); however, within this model, there were no significant main or interaction effects. Models predicting aDMN connectivity with other prefrontal regions were not significant (BA6: *p* = 0.44; BA8: *p* = 0.23; BA9: *p* = 0.19; see Table 2).

**Table 2:** Summary of regression analyses for the connectivity between left prefrontal regions and anterior default mode network.

**Variable**	***B***	***p***	**95% Confidence interval**	
**CONNECTIVITY BETWEEN LEFT BA6 AND ANTERIOR DMN**				
Overall model		0.44		
APOE4	−0.093	0.75	−0.681	0.495
Amyloid	−0.030	0.87	−0.396	0.337
Sex	−0.091	0.15	−0.216	0.033
APOE4^*^amyloid	−0.008	0.98	−0.728	0.710
APOE4^*^sex	0.036	0.84	−0.306	0.379
Amyloid^*^sex	0.047	0.68	−0.180	0.273
APOE4^*^amyloid^*^sex	−0.019	0.93	−0.447	0.408
Age	0.005	0.10	−0.0009	0.011
Education	−0.001	0.90	−0.019	0.016
**CONNECTIVITY BETWEEN LEFT BA44 AND ANTERIOR DMN**				
Overall model		0.05		
APOE4	−0.378	0.20	−0.964	0.208
Amyloid	−0.265	0.15	−0.630	0.010
Sex	−0.100	0.11	−0.224	0.024
APOE4^*^amyloid	0.260	0.47	−0.456	0.976
APOE4^*^sex	0.156	0.37	−0.185	0.497
Amyloid^*^sex	0.178	0.12	−0.047	0.404
APOE4^*^amyloid^*^sex	−0.163	0.45	−0.589	0.263
Age	0.005	0.09	−0.0009	0.011
Education	0.007	0.44	−0.010	0.024
**CONNECTIVITY BETWEEN LEFT BA8 AND ANTERIOR DMN**				
Overall model		0.23		
APOE4	0.169	0.50	−0.330	0.669
Amyloid	0.252	0.11	−0.059	0.563
Sex	−0.038	0.48	−0.144	0.067
APOE4^*^amyloid	−0.507	0.10	−1.118	0.104
APOE4^*^sex	−0.126	0.39	−0.417	0.165
Amyloid^*^sex	−0.160	0.10	−0.352	0.032
APOE4^*^amyloid^*^sex	0.287	0.12	−0.076	0.650
Age	0.000	0.99	−0.005	0.005
Education	−0.003	0.69	−0.018	0.012
**CONNECTIVITY BETWEEN LEFT BA9 AND ANTERIOR DMN**				
Overall model		0.19		
APOE4	0.055	0.83	−0.450	0.559
Amyloid	0.351	0.03	0.037	0.666
Sex	−0.018	0.74	−0.125	0.089
APOE4^*^amyloid	−0.572	0.07	−1.188	0.045
APOE4^*^sex	−0.081	0.59	−0.374	0.213
Amyloid^*^sex	−0.224	0.02	−0.418	−0.030
APOE4^*^amyloid^*^sex	0.411	0.03	0.045	0.777
Age	−0.002	0.52	−0.007	0.004
Education	−0.001	0.89	−0.016	0.014

* *Connectivity in Fisher's z. APOE4, Apolipoprotein ε4 allele; BA, Brodmann area; DMN, Default Mode Network*.

For the pDMN, the overall model predicting BA9/pDMN connectivity was significant (*p* = 0.003), but the 3-way sex by A+/– by *APOE* ε*4* interaction was not (*p* = 0.09). Main effects of age (*p* = 0.002), sex (*p* = 0.03), and education (*p* = 0.04) were noted, with women showing greater connectivity and older and more educated individuals showing less connectivity. Models predicting connectivity of other prefrontal regions to pDMN were not significant (BA6: *p* = 0.93; BA44: *p* = 0.76; BA8: *p* = 0.21; Table 3).

**Table 3:** Summary of regression analyses for connectivity between left prefrontal regions and the posterior default mode network.

**Variable**	***B***	***p***	**95% Confidence interval**	
**CONNECTIVITY BETWEEN LEFT BA6 AND POSTERIOR DMN**				
Overall model		0.93		
APOE4	−0.232	0.36	−0.726	0.263
Amyloid	0.030	0.16	−0.279	0.338
Sex	0.007	0.90	−0.098	0.111
APOE4^*^amyloid	0.254	0.41	−0.350	0.858
APOE4^*^sex	0.115	0.43	−0.173	0.403
Amyloid^*^sex	0.022	0.82	−0.168	0.212
APOE4^*^amyloid^*^sex	−0.153	0.40	−0.513	0.206
Age	−0.001	0.59	−0.007	0.004
Education	0.003	0.67	−0.012	0.018
**CONNECTIVITY BETWEEN LEFT BA44 AND POSTERIOR DMN**				
Overall model		0.76		
APOE4	−0.343	0.15	−0.805	0.120
Amyloid	−0.050	0.73	−0.338	0.239
Sex	0.035	0.48	−0.063	0.133
APOE4^*^amyloid	0.464	0.11	−0.102	1.029
APOE4^*^sex	0.167	0.22	−0.102	0.436
Amyloid^*^sex	0.025	0.78	−0.153	0.203
APOE4^*^amyloid^*^sex	−0.265	0.12	−0.602	0.071
Age	−0.0007	0.78	−0.005	0.004
Education	0.006	0.39	−0.008	0.020
**CONNECTIVITY BETWEEN LEFT BA8 AND POSTERIOR DMN**				
Overall model		0.20		
APOE4	0.117	0.71	−0.499	0.734
Amyloid	0.181	0.35	−0.203	0.565
Sex	0.082	0.22	−0.048	0.212
APOE4^*^amyloid	−0.197	0.61	−0.951	0.557
APOE4^*^sex	−0.077	0.67	−0.435	0.282
Amyloid^*^sex	−0.169	0.16	−0.406	0.069
APOE4^*^amyloid^*^sex	0.168	0.46	−0.280	0.616
Age	−0.008	0.02	−0.014	−0.001
Education	−0.015	0.12	−0.033	0.004
**CONNECTIVITY BETWEEN LEFT BA9 AND POSTERIOR DMN**				
Overall model		0.003		
APOE4	0.338	0.30	−0.304	0.979
Amyloid	0.252	0.21	−0.148	0.652
Sex	0.154	0.03	0.019	0.290
APOE4^*^amyloid	−0.467	0.24	−1.251	0.317
APOE4^*^sex	−0.230	0.23	−0.603	0.143
Amyloid^*^sex	−0.229	0.07	−0.475	0.018
APOE4^*^amyloid^*^sex	0.405	0.09	−0.061	0.872
Age	−0.011	0.002	−0.018	−0.004
Education	−0.020	0.04	−0.039	0.001

* *Connectivity in Fisher's z. APOE4, Apolipoprotein ε4 allele; BA, Brodmann area; DMN, Default Mode Network*.

Secondary analyses within NC showed the model for BA9/pDMN was significant (*p* = 0.008) and the uncorrected three-way sex by A+/– by *APOE* ε*4* interaction was also significant (*p* = 0.03). The interaction was not significant after multiple comparison correction; however, parsing the interaction suggested an *APOE* ε*4* by A+/– effect on connectivity only in women (*p* = 0.005) and not men (*p* = 0.56), with lowest connectivity in A–/ε4+ women.

The secondary analysis within eMCI individuals showed the model was not significant for BA9/pDMN (*p* = 0.15).

### Sex Moderates aDMN/pDMN Connectivity Across Diagnoses

**Figure 2:**
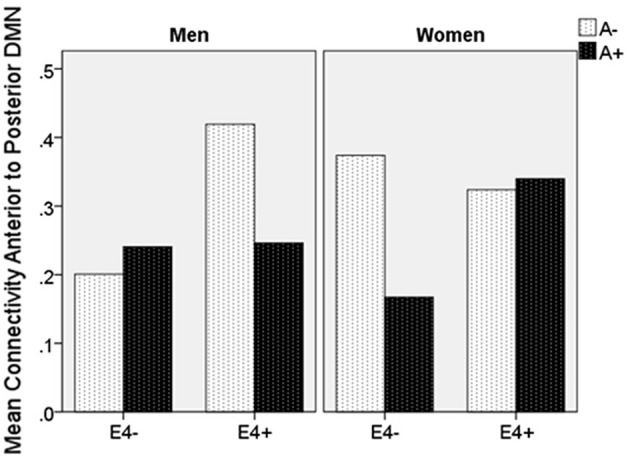
Mean resting state functional connectivity by sex, APOE ε4 carrier status, and amyloid status. APOE ε4: Apolipoprotein 4. ε4(+/−) indicates presence or absence of gene. A(+/−) indicates presence or absence of positive amyloid PET scan. Units are Pearson's r-values.

Moderation regression results showed a significant overall model predicting connectivity from aDMN to pDMN (*p* = 0.0003). This model also evidenced a significant uncorrected 3-way interaction effect of sex, A+/–, and *APOE* ε*4* status on connectivity (*p* = 0.01; significant after correction for multiple comparisons correction). Specifically, there was an *APOE* ε*4* by A+/– interaction effect on aDMN/pDMN connectivity only in women (*p* = 0.04; not significant after multiple comparison correction) and not men (*p* = 0.11). Parsing this interaction showed a trend toward *APOE* ε*4* effect on aDMN/pDMN connectivity in women who were A+ (*p* = 0.07), and not in those who were A– (*p* = 0.26). Specifically, A+, ε4+ women showed more positive aDMN/pDMN connectivity. For men, the trend-level result suggested the opposite pattern (i.e., A– [*p* = 0.07] rather than A+ [*p* = 0.93] men showing a relationship of *APOE* ε*4* to greater aDMN/pDMN connectivity). This model also showed a main effect of age (*p* < 0.001), with older individuals showing less connectivity (Table 4; Figure 2).

**Table 4:** Summary of regression analyses for connectivity between anterior and posterior default mode network.

**Variable**	**B**	***p***	**95% Confidence interval**	
**CONNECTIVITY BETWEEN ANTERIOR AND POSTERIOR DMN**				
Overall model		0.0003		
APOE4	0.652	0.03	0.047	1.256
Amyloid	0.373	0.05	−0.004	0.750
Sex	0.167	0.01	0.040	0.295
APOE4^*^amyloid	−0.851	0.02	−1.591	−0.112
APOE4^*^sex	−0.385	0.03	−0.737	−0.033
Amyloid^*^sex	−0.305	0.01	−0.538	−0.072
APOE4^*^amyloid^*^sex	0.569	0.01	0.129	1.008
Age	−0.012	0.0002	−0.019	−0.006
Education	−0.018	0.06	−0.036	0.0004

* *Connectivity in Fisher's Z*.

Secondary analyses showed that for NC, aDMN/pDMN connectivity findings were similar to those for the full sample, though did not pass multiple comparison correction (overall model *p* = 0.0004; 3-way interaction *p* = 0.014; interaction of *APOE* ε*4* by A+/– significant in women [*p* = 0.02] and not men [*p* = 0.20]). The model for MCI was not significant (*p* = 0.09).

### aDMN/pDMN Connectivity Relates to Verbal Memory

**Figure 3:**
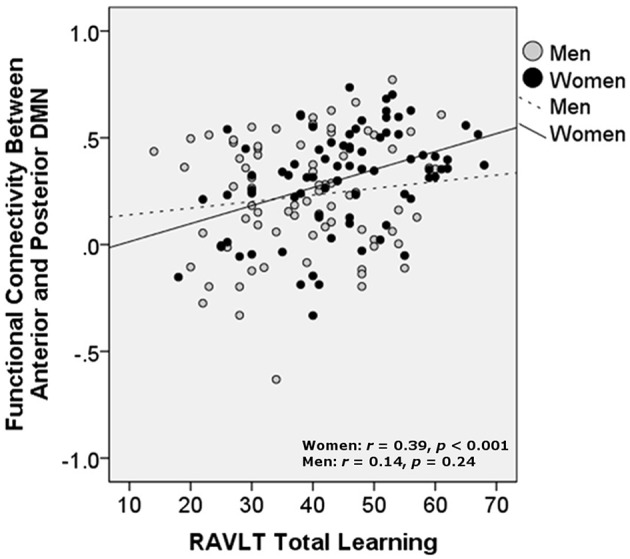
Relationship of verbal learning with resting state functional connectivity between the anterior and posterior DMN, by sex. DMN, Default mode network; RAVLT, Rey Auditory Verbal Learning Test. Units are Pearson's r-values.

Partial correlations controlling for age and education showed that increased aDMN/pDMN connectivity related to better verbal learning in women (*p* = 0.006; simple Pearson correlation values: *r* = 0.39, *p* < 0.001) and not men (*p* = 0.18; simple Pearson correlation values: *r* = 0.14, *p* = 0.24). Increased aDMN /pDMN connectivity did not significantly relate to delayed recall after partialling out age and education (women: *p* = 0.13; men: *p* = 0.43; Figure 3). aDMN/pDMN connectivity was not correlated with education alone (*p* = 0.53).

## Discussion

The current investigation showed that sex did not moderate the impact of *APOE* ε*4* genotype and amyloid status on left prefrontal/DMN connectivity across NC and MCI cohorts. However, sex did moderate the impact of *APOE* ε*4* genotype and amyloid positivity on functional connectivity between anterior and posterior DMN. In particular, aDMN/pDMN connectivity was greater in the presence of an *APOE* ε*4* genotype, but only in women with a positive amyloid scan. In other words, connectivity was highest in those with most AD risk and burden. Interestingly, for women only, greater aDMN/pDMN connectivity was correlated with better verbal learning. Men showed trend-level results suggesting that *APOE* ε*4* genotype was associated with greater connectivity only for individuals who were amyloid negative—the opposite pattern to women.

The primary hypotheses of this investigation were not supported, suggesting that women's verbal memory reserve does not relate to left prefrontal functional connectivity with the DMN, which may underlie more general cognitive reserve. In our sample, left prefrontal and DMN connectivity were also not correlated with education, a typical marker of general cognitive reserve, and this may explain our overall negative finding. Lack of findings may also relate to sample-size or methodological choices (i.e., excluding right prefrontal and bilateral temporal cortex; defining DMN on a subject-specific basis but left PFC based on the Brodmann atlas). Another factor to consider is that work has shown that beyond cognitive reserve and underlying neural reserve, complex genetic interactions may have a role in resilience to AD. Assessing the wide array of genetic contributors, some of which are known to differ by sex (43–45), was beyond the scope of this analysis, but may be key in understanding the neural basis of women's memory reserve in AD. Despite these caveats, secondary analyses suggested that BA9/pDMN connectivity may be moderated by sex in individuals with normal cognition. Given reduced power in our secondary analyses, this finding warrants follow-up in larger samples.

Our finding that sex moderated the impact of A+ and *APOE* ε*4* status on connectivity between anterior and posterior DMN, and that greater aDMN/pDMN connectivity related to better verbal learning in women overall, will also require follow-up. This pattern could suggest reserve in verbal learning or compensation in women, and this could be further evaluated using longitudinal approaches. In the context of prior work showing an *APOE* ε*4* by sex interaction in the pDMN resulting in less global pDMN connectivity in ε4 positive healthy women (27), the present results could suggest that specific aspects of pDMN connectivity are maintained or increased in women at highest risk, while global pDMN connectivity declines. Our findings for aDMN/pDMN connectivity appear to be driven by the NC group, which could be consistent with early resilience to overall DMN dysfunction over time. At the same time, tighter aDMN/pDMN coupling in the most at risk individuals is unlikely to be advantageous in the long term [i.e., (26)] and may play a role in women's steeper decline after the MCI stage. Interestingly, the pattern in ε4+/A+ women was opposite of the pattern in men and also opposite of the age effect, which predicted less connectivity. Given the age of our sample, our age findings are consistent with recent studies in healthy aging (46), but complex interaction of age and other risk factors may warrant additional assessment. Of note, the lack of interaction in men appears to be due to amyloid positivity having an effect on connectivity that does not differ by ε4 status. In contrast, a main effect of ε4 allele presence on greater connectivity in men is suggested. While this could not be directly assessed in the current model, it deserves attention in future investigations.

Limitations of the present study include the high education level of the ADNI sample and relatively small sample size for examination of complex interactive effects. Future efforts should aim to replicate these findings in larger and longitudinal samples, and to carefully consider effects of diagnosis. It will also be key to examine functional connectivity at rest of other regions suggested to be important for cognitive reserve, such as the right prefrontal cortex and left inferior temporal cortex. In addition, the present analysis included a small number of individuals with SMC but no cognitive impairment in the normal control group. As SMC have been associated with cognitive decline, this analytical choice may have impacted our findings, and it will be important to further assess the SMC group independently in the future. Moreover, given the relatively large number of variables of interest in the present analysis, diagnostic group was not treated formally as a measure of interest in the primary analysis. This is a limitation that warrants further assessment. Beyond resting-state investigations, it may also be informative to examine sex-based reserve correlates in task-based fMRI, focusing on memory.

Overall, the strengths of the present investigation lie in its novel integration of the otherwise separate research literatures on general cognitive reserve and sex-based memory reserve as assessed by functional resting state connectivity. We use a relatively large and well-characterized sample to show that women's memory reserve may be supported in part by complex interplay between networks typically most active at rest and prefrontal brain regions. These findings lend support for the continued importance of considering sex as a factor of importance in AD neural correlates. Better understanding of these correlates may in turn be helpful in delineating targets for treatment.

## Data Availability

The datasets analyzed for this study can be found at the Alzheimer's Disease Neuroimaging Initiative website (ADNI; http://adni-info.org).

## Ethics Statement

This study was carried out in accordance with the recommendations of ADNI local site Institutional Review Boards, with written informed consent from all subjects. All subjects gave written informed consent in accordance with the Declaration of Helsinki. The study was approved by each participating ADNI site's local Institutional Review Boards.

## Author Contributions

JCa designed the study, interpreted data, and prepared the manuscript. XZ analyzed and interpreted data. ML acquired data and prepared the manuscript. JCu and SB provided critical revision of the manuscript for intellectual content. DC provided critical input to study design and data interpretation, and also provided critical revision of the manuscript for intellectual content. All authors read and approved the final manuscript.

### Conflict of Interest Statement

JCu has provided consultation to Acadia, Accera, Actinogen, Alkahest, Allergan, Alzheon, Avanir, Axsome, BiOasis Technologies, Biogen, Diadem, EIP Pharma, Eisai, Genentech, Green Valley, Grifols, Hisun, Idorsia, Kyowa Kirin, Lilly, Lundbeck, Merck, Otsuka, Proclara, QR, Resverlogix, Roche, Samus, Samumed, Sunovion, Suven, Takeda, Teva, Toyama, and United Neuroscience pharmaceutical and assessment companies. JCu acknowledges funding from the National Institute of General Medical Sciences (Grant: P20GM109025) and support from Keep Memory Alive. The remaining authors declare that the research was conducted in the absence of any commercial or financial relationships that could be construed as a potential conflict of interest.

## References

[1] Herbert LE Weuve J Scherr PA Evans DA Alzheimer disease in the United States (2010-2050) estimated using the 2010 Census. Neurology. (2013) 80:1778–83. 10.1212/WNL.0b013e31828726f5 23390181 PMC3719424

[2] Nebel RA Aggarwal NT Barnes LL Gallagher A Goldstein JM Kantraci K. Understanding the impact of sex and gender in Alzheimer's disease: a call to action. Alzheimers Dement. (2018) 14:1171–83. 10.1016/j.jalz.2018.04.008 29907423 PMC6400070

[3] Buckley RF Mormino EC Amariglio RE Properzi MJ Rabin JS Lim YY. Sex, amyloid, and *APOE* ε*4* and risk of cognitive decline in preclinical Alzheimer's disease: findings from three well-characterized cohorts. Alzheimers Dement. (2018) 14:1193–203. 10.1016/j.jalz.2018.04.010 29803541 PMC6131023

[4] Koran MEI Wagener M Hohman TJ. Sex differences in the association between AD biomarkers and cognitive decline. Brain Imaging Behav. (2017) 11:205–13. 10.1007/s11682-016-9523-8 26843008 PMC4972701

[5] Sundermann EE Biegon A Rubin LH Lipton RB Landau S Maki PM. Does the female advantage in verbal memory contribute to underestimating Alzheimer's disease pathology in women versus men? J Alzheimer's Dis. (2017) 56:947–57. 10.3233/JAD-160716 28106548 PMC7644197

[6] Caldwell JZK Berg JL Cummings JL Banks SJ. Sex moderates theimpact of diagnosis and amyloid PET positivity on hippocampal subfield volume. J Alzheimer's Dis. (2018) 64:79–89. 10.3233/JAD-180028 29865063 PMC6004904

[7] Sundermann EE Biegon A Rubin LH Lipton RB Mowrey W Landau S. Better verbal memory in women than men in MCI despite similar levels of hippocampal atrophy. Neurology. (2016) 86:1368–76. 10.1212/WNL.0000000000002570 26984945 PMC4831033

[8] Sundermann EE Maki PM Rubin LH Lipton RB Landau S Biegon A. Female advantage in verbal memory Evidence of sex-specific cognitive reserve. Neurology. (2016) 87:1916–24. 10.1212/WNL.0000000000003288 27708128 PMC5100712

[9] McCarrey AC An Y Kitner-Triolo MH Ferrucci L Resnick SM. Sex differences in cognitive trajectories in clinicallynormal older adults. Psychol Aging. (2016) 31:166–75. 10.1037/pag0000070 26796792 PMC4783196

[10] Stern Y. Cognitive reserve in ageing and Alzheimer's disease. Lancet Neurol. (2012) 11:1006–2. 10.1016/S1474-4422(12)70191-6 23079557 PMC3507991

[11] Mungas D Gavett B Fletcher E Farias ST DeCarli C Reed B. Education amplifies brain atrophy effect on cognitive decline: implications for cognitive reserve. Neurobiol Aging. (2018) 68:142–50. 10.1016/j.neurobiolaging.2018.04.002 29798764 PMC5993638

[12] Caldwell JZK Berg J-L Cummings JL Banks SJ. Moderating effects of sex on the impact of diagnosis and amyloid positivity on verbal memory and hippocampal volume. Alzheimers Res Ther. (2017) 9:72. 10.1186/s13195-017-0300-8 28899422 PMC5596932

[13] Braak H Braak E Neuropathological staging of Alzheimer-related changes. Acta Neuropathol. (1991) 82:239–59. 10.1007/BF00308809 1759558

[14] Franzmeier N Caballero MAA Taylor ANW Simon-Vermot L Buerger K Ertl-Wagner B. Resting-state global functional connectivity as a biomarker of cognitive reserve in mild cognitive impairment. Brain Imaging Behav. (2017) 11:368–82. 10.1007/s11682-016-9599-1 27709513

[15] Franzmeier N Buerger K Teipel S Stern Y Dichgans M Ewers M. Cognitive reserve moderates the association between functional network anti-correlations and memory in MCI. Neurobiol Aging. (2017) 50:152–62. 10.1016/j.neurobiolaging.2016.11.013 28017480

[16] Franzmeier N Gottler J Grimmer T Drzezga A Araque-Caballero MA Simon-Vermot L. Resting-state connectivity of the left frontal cortex to the default mode and dorsal attention network supports reserve in mild cognitive impairment. Front Aging Neurosci. (2017) 9:264. 10.3389/fnagi.2017.00264 28824423 PMC5545597

[17] Raichle ME MacLeod AM Snyder AZ Powers WJ Gusnard DA Shulman GL A default mode of brain function. Proc Natl Acad Sci USA. (2001) 98:676–82. 10.1073/pnas.98.2.676 11209064 PMC14647

[18] Buckner RL Andrews-Hanna JR Schacter DL. The brain's default network: anatomy, function, and relevance to disease. Ann NY Acad Sci. (2008) 1124:1–38. 10.1196/annals.1440.011 18400922

[19] Greicius MD Srivastava G Reiss AL Menon V. Default-mode network activity distinguishes Alzheimer's disease from healthy aging: evidence from functional MRI. Proc Natl Acad Sci USA. (2004) 101:4637–2. 10.1073/pnas.0308627101 15070770 PMC384799

[20] Sorg C Riedl V Muhlau M Calhoun VD Eichele T Laer L. Selective changes of resting state networks in individuals at risk for Alzheimer's disease. Proc Natl Acad Sci USA. (2007) 104:18760–5. 10.1073/pnas.0708803104 18003904 PMC2141850

[21] Damoiseaux JS Prater KE Miller BL Greicius MD. Functional connectivity tracks clinical deterioration in Alzheimer's disease. Neurobiol Aging. (2012) 33:e19–30. 10.1016/j.neurobiolaging.2011.06.024 21840627 PMC3218226

[22] Wang Z Liang P Jia X Jin G Song H Han Y. The baseline and longitudinal changes of PCC connectivity in mild cognitive impairment: a combined structure and resting-state fMRI study. PLoS ONE. (2012) 7:e36838. 10.1371/journal.pone.0036838 22629335 PMC3356348

[23] Hedden T Van Dijk KR Becker JA Mehta A Sperling RA Johnson KA. Disruption of functional connectivity in clinically normal older adults harboring amyloid burden. J Neurosci. (2009) 29:12686–94. 10.1523/JNEUROSCI.3189-09.2009 19812343 PMC2808119

[24] Sheline YI Raichle ME Snyder AZ Morris JC Head D Wang S. Amyloid plaques disrupt resting state default mode network connectivity in cognitively normal elderly. Biol Psychiatry. (2010) 67:584–7. 10.1016/j.biopsych.2009.08.024 19833321 PMC2829379

[25] Sheline YI Morris JC Snyder AZ Price JL Yan Z D'Angelo G *APOE* ε*4* allele disrupts resting state fMRI connectivity in the absence of amyloid plaques or decreased CSF Aβ42. J Neurosci. (2010) 30:17035–40. 10.1523/JNEUROSCI.3987-10.2010 21159973 PMC3023180

[26] Jones DT Knopman DS Gunter JL Graff-Radford J Vemuri P Boeve BF. Cascading network failure across the Alzheimer's disease spectrum. Brain. (2016) 139:547–62. 10.1093/brain/awv338 26586695 PMC4805086

[27] Damoiseaux JS Seeley WW Zhou J Shirer WR Coppola G Karydas A. Gender modulates the *APOE* ε*4* effect in healthy older adults: convergent evidence from functional brain connectivity and spinal fluid tau levels. J Neurosci. (2012) 32:8254–62. 10.1523/JNEUROSCI.0305-12.2012 22699906 PMC3394933

[28] Smith EE Jonides J Koeppe RA. Dissociating verbal and spatial working memory using PET. Cereb Cortex. (1996) 6:11–20. 10.1093/cercor/6.1.11 8670634

[29] van Veluw SJ Sawyer EK Clover L Cousijn H De Jager C Esiri MM. Prefrontal cortex cytoarchitechture in normal aging and Alzheimer's disease: a relationship with IQ. Brain Struct Funct. (2012) 217:797–808. 10.1007/s00429-012-0381-x 22302432

[30] Morbelli S Perneczky R Drzezga A Frisoni GB Caroli A van Berckel BN. Metabolic networks underlying cognitive reserve in prodromal Alzheimer disease: a European Alzheimer disease consortium project. J Nucl Med. (2013) 54:894–902. 10.2967/jnumed.112.113928 23591639

[31] Brosnan MB Demaria G Petersen A Dockree PM Robertson IH Wiegand I. Plasticity of the right-lateralized cognitive reserve network in ageing. Cereb Cortex. (2018) 28:1749–59. 10.1093/cercor/bhx085 28444373

[32] Robertson IH. Right hemisphere role in cognitive reserve. Neurobiol Aging. (2014) 35:1375–85. 10.1016/j.neurobiolaging.2013.11.028 24378088

[33] Bauckneht M Chincarini A Piva R Arnaldi D Girtler N Massa F. Metabolic correlates of reserve and resilience in MCI due to Alzheimer's Disease (AD). Alzheimers Res Ther. (2018) 10:35. 10.1186/s13195-018-0366-y 29615111 PMC5883593

[34] Folstein MF Folstein SE McHugh PR “Mini-mental state.” A practical method for grading the cognitive state of patients for the clinician. J Psychiatr Res. (1975) 12:189–98. 1202204 10.1016/0022-3956(75)90026-6

[35] Morris JC. Clinical dementia rating: current version and scoring rules. Neurology. (1993) 43:2412–4. 10.1212/WNL.43.11.2412-a 8232972

[36] Petersen RC Aisen PS Beckett LA Donohue MC Gamst AC Harvey DJ. Alzheimer's Disease Neuroimaging Initiative (ADNI): clinical characterization. Neurology. (2010) 74:201–9. 10.1212/WNL.0b013e3181cb3e25 20042704 PMC2809036

[37] Rey A L'examen Clinique En Psychologie. Paris: Presses Universitaires de France (1964).

[38] Power JD Mitra A Laumann TO Snyder AZ Schlaggar BL Petersen SE. Methods to detect, characterize, and remove motion artifact in resting state fMRI. Neuroimage. (2014) 84:320–41. 10.1016/j.neuroimage.2013.08.048 23994314 PMC3849338

[39] Hyvärinen A Karhunen J Oja E Independent Component Analysis. John Wiley & Sons, Inc. (2002).

[40] Du Y Fan Y. Group information guided ICA for fMRI data analysis. Neuroimage. (2013) 69:157–97. 10.1016/j.neuroimage.2012.11.008 23194820

[41] Hayes AF Introduction to Mediation, Moderation, and Conditional Process Analysis: A Regression Based-Approach. New York, NY: Guillford Press (2013).

[42] IBM Corp Released IBM SPSS Statistics for Windows, Version 23.0. Armonk, NY: IBM Corp (2015).

[43] Hohman TJ McLaren DG Mormino EC Gifford KA Libon DJ Jefferson AL. Asymptomatic Alzheimer disease: defining resilience. Neurology. (2016) 87:2443–50. 10.1212/WNL.0000000000003397 27815399 PMC5177674

[44] Hohman TJ Dumitrescu L Cox NJ Jefferson AL. Genetic resilience to amyloid related cognitive decline. Brain Imaging Behav. (2017) 11:401–9. 10.1007/s11682-016-9615-5 27743375 PMC5392179

[45] Deming Y Dumitrescu L Barnes LL Thambisetty M Kunkle B Gifford KA. Sex-specific genetic predictors of Alzheimer's disease biomarkers. Acta Neuropathol. (2018) 136:857–72. 10.1007/s00401-018-1881-4 29967939 PMC6280657

[46] Staffaroni AM Brown JA Casaletto KB Elahi FM Deng J Neuhaus J. The longitudinal trajectory of default mode network connectivity in healthy older adults varies as a function of age and is associated with changes in episodic memory and processing speed. J Neurosci. (2018) 38:2809–17. 10.1523/JNEUROSCI.3067-17.2018 29440553 PMC5852659

