# Supplementary material for: Sex Moderates Amyloid and Apolipoprotein ε4 Effects on Default Mode Network Connectivity at Rest
Source: Front Neurol. 2019 Aug 20;10:900. doi: 10.3389/fneur.2019.00900 (PMC6710397; doi:10.3389/fneur.2019.00900)
Supplement: Supplementary file 1 [file Table_1.docx]

Table S1: Participant Demographics and Group Differences by Sex (NC)

|  | Male | Female | Group difference (p-value) |
| --- | --- | --- | --- |
| Number of subjects | 46 | 47 |  |
| Amyloid status | 15 positive | 22 positive | 0.161 |
| APOE4 | 10 | 13 | 0.508 |
| Age | 77.96$\pm$7.08 | 75.73$\pm$7.03 | 0.131 |
| Handedness (Right/Left) | 41/5 | 42/5 | 0.971 |
| Years of Education | 16.93$\pm$2.18 | 15.91$\pm$2.70 | 0.042 |
| RMS motion (mm) | 0.32$\pm$ 0.23 | 0.29 $\pm$ 0.23 | 0.504 |

APOE4: Apolipoprotein Ɛ4 allele; RMS: Root Mean Squared.

*p*-values reflect significance of Mann-Whitney tests.
