# Supplementary material for: Sex Moderates Amyloid and Apolipoprotein ε4 Effects on Default Mode Network Connectivity at Rest
Source: Front Neurol. 2019 Aug 20;10:900. doi: 10.3389/fneur.2019.00900 (PMC6710397; doi:10.3389/fneur.2019.00900)
Supplement: Supplementary file 2 [file Table_2.docx]

Table S2: Participant Demographics and Group Differences by Sex (early Mild Cognitive Impairment)

|  | Male | Female | Group difference (p-value) |
| --- | --- | --- | --- |
| Number of subjects | 33 | 32 |  |
| Amyloid status | 18 positive | 16 positive | 0.714 |
| APOE4 | 14 | 13 | 0.883 |
| Age | 74.55$\pm$5.27 | 71.84$\pm$8.29 | 0.119 |
| Handedness (Right/Left) | 31/2 | 30/2 | 0.975 |
| Years of Education | 16.36$\pm$2.73 | 15.66$\pm$2.81 | 0.307 |
| RMS motion (mm) | 0.33$\pm$ 0.26 | 0.26 $\pm$ 0.20 | 0.200 |

APOE4: Apolipoprotein Ɛ4 allele; RMS: Root Mean Squared.

*p*-values reflect significance of Mann-Whitney tests.
