# Supplementary material for: Sex Moderates Amyloid and Apolipoprotein ε4 Effects on Default Mode Network Connectivity at Rest
Source: Front Neurol. 2019 Aug 20;10:900. doi: 10.3389/fneur.2019.00900 (PMC6710397; doi:10.3389/fneur.2019.00900)
Supplement: Supplementary file 3 [file Table_3.docx]

Table S3: Participant Demographics and Group Differences by Sex (Full Sample)

|  |  | Amyloid Negative | | |  | Amyloid Positive | | |
| --- | --- | --- | --- | --- | --- | --- | --- | --- |
|  |  |  |  |  |  |  |  |  |
|  |  | CN |  | eMCI |  | CN |  | eMCI |
| Number of subjects |  | 56 |  | 31 |  | 37 |  | 34 |
| APOE4 |  | 7^b^ |  | 6^c^ |  | 16 |  | 21 |
| Age |  | 76.43±7.39^a^ |  | 71.53±7.04 |  | 77.44±6.71 |  | 74.76±6.70 |
|  |  |  |  |  |  |  |  |  |
| Handedness (Right/Left) |  | 51/5 |  | 28/3 |  | 32/5 |  | 33/1 |
| Years of Education |  | 16.98±1.96 ^b^ |  | 16.35±2.78 |  | 15.57±2.91 |  | 15.71±2.77 |
|  |  |  |  |  |  |  |  |  |
| RMS motion (mm) |  | 0.29±0.20 |  | 0.29±0.23 |  | 0.39±0.33^c^ |  | 0.26±0.19 |
|  |  |  |  |  |  |  |  |  |
| RAVLT immediate |  | 43.55±10.66 |  | 39.80±12.76 |  | 42.28±10.59 |  | 39.68±12.61 |
| RAVLT Delay |  | 6.86±1.96 |  | 6.10±4.47 |  | 6.25±3.86 |  | 4.53±4.60 |
|  |  |  |  |  |  |  |  |  |

APOE4: Apolipoprotein Ɛ4 allele; CN: Cognitively Normal; eMCI: early Mild Cognitive Impairment; RAVLT: Rey Auditory Verbal Learning Test; RMS: Root Mean Squared.

^a^ Mann-Whitney test shows significant difference from Amyloid Negative eMCI

^b^ Mann-Whitney test shows significant difference from Amyloid Positive CN

^c^ Mann-Whitney test shows significant difference from Amyloid Positive eMCI
