# Supplementary material for: Sex Moderates Amyloid and Apolipoprotein ε4 Effects on Default Mode Network Connectivity at Rest
Source: Front Neurol. 2019 Aug 20;10:900. doi: 10.3389/fneur.2019.00900 (PMC6710397; doi:10.3389/fneur.2019.00900)
Supplement: Supplementary file 4 [file Table_4.docx]

Table S4: Descriptive statistics for connectivity in secondary analyses by diagnosis

| Connectivity between BA9 and posterior DMN | | | | | | | | |
| --- | --- | --- | --- | --- | --- | --- | --- | --- |
|  | Cognitively Normal | | | | | | | |
|  |  | Men | | |  | Women | | |
| Variable |  | Amyloid - |  | Amyloid + |  | Amyloid - |  | Amyloid + |
| APOE 4- |  | 0.121(0.346) |  | 0.175(0.242) |  | 0.386(0.212) |  | 0.149(0.320) |
| APOE 4+ |  | 0.319(0.034) |  | 0.247(0.153) |  | 0.121(0.322) |  | 0.337(0.201) |
|  |  |  |  |  |  |  |  |  |
|  | eMCI | | | | | | | |
|  |  | Men | | |  | Women | | |
| Variable |  | Amyloid - |  | Amyloid + |  | Amyloid - |  | Amyloid + |
| APOE 4- |  | 0.215(0.267) |  | 0.156(0.319) |  | 0.171(0.308) |  | 0.130(0.176) |
| APOE4+ |  | 0.165(0.336) |  | 0.199(0.202) |  | 0.449(0.070) |  | 0.358(0.165) |
|  |  | | | | | | | |
| Connectivity between anterior DMN and posterior DMN | | | | | | | | |
|  | Cognitively Normal | | | | | | | |
|  |  | Men | | |  | Women | | |
| Variable |  | Amyloid - |  | Amyloid + |  | Amyloid - |  | Amyloid + |
| APOE 4- |  | 0.168(0.304) |  | 0.191(0.320) |  | 0.391(0.193) |  | 0.189(0.272) |
| APOE 4+ |  | 0.560(0.049) |  | 0.346 (0.147) |  | 0.226(0.345) |  | 0.361(0.096) |
|  |  |  |  |  |  |  |  |  |
|  | eMCI | | | | | | | |
|  |  | Men | | |  | Women | | |
| Variable |  | Amyloid - |  | Amyloid + |  | Amyloid - |  | Amyloid + |
| APOE 4- |  | 0.275(0.234) |  | 0.299(0.333) |  | 0.345(0.225) |  | 0.124(0.244) |
| APOE 4+ |  | 0.278(0.332) |  | 0.179(0.250) |  | 0.447(0.175) |  | 0.321(0.239) |

APOE4: Apolipoprotein Ɛ4 allele; BA: Brodmann Area; DMN: Default Mode Network; eMCI: early

Mild Cognitive Impairment
